# A Neutraceutical by Design: The Clinical Application of Curcumin in Colonic Inflammation and Cancer

**DOI:** 10.6064/2012/757890

**Published:** 2012-09-03

**Authors:** D. Soni, B. Salh

**Affiliations:** Division of Gastroenterology, Department of Medicine, The University of British Columbia, Vancouver, BC, Canada V5Z 1M9

## Abstract

Unquestionably, the natural food additive curcumin, derived from the colorful spice turmeric used in many Asian cuisines, possesses a diverse array of biological activities. These range from its anti-inflammatory, antineoplastic, and metabolic modifying properties to surprising roles in disorders ranging from Alzheimer's disease to cystic fibrosis. Its effects on growth factor receptors, signaling molecules, and transcription factors, together with its epigenetic effects are widely considered to be extraordinary. These pleiotropic attributes, coupled with its safety even when used orally at well over 10 g/day, are unparalleled amongst pharmacological agents. However, there is one drawback; apart from the luminal gastrointestinal tract where its pharmacology predicts that reasonable drug levels can be attained, its broader use is hampered by its poor solubility and hence near undetectable plasma levels. Medicinal chemistry and nanotechnology have resulted in the generation of compounds where the modified drug or its delivery system has improved matters such that this shortcoming has been addressed to some extent, with the surprising finding that it remains safe to use. It is predicted that either the parental compound or its derivatives may eventually find a place in the therapeutic management protocols of several conditions.

## 1. Background

Gastrointestinal disorders in general are probably the commonest reasons for patients seeking medical attention. Whilst many of these are short-lived and due to minor infectious illnesses and food intolerances, there are still a significant number of more serious and chronic problems that demand ongoing or more involved care [1]. This is especially true of gastrointestinal cancers, that include colorectal cancer and chronic inflammatory diseases such as Crohn's disease and Ulcerative colitis.

Colorectal cancer is one of the leading causes of morbidity and mortality worldwide; contributing to 8% of all cancer deaths, it is the third most common cancer in women and fourth most common cancer in men [2, 3]. An estimated 1.2 million new colorectal cancer cases are detected annually, accounting for approximately 630 000 deaths per year. Patients with inflammatory bowel disease (IBD) have a strong predisposing risk for colorectal cancer [4]. IBD is a broad term, comprising Crohn's disease (CD) and Ulcerative colitis (UC). The unification of the two under a common banner rests on a mutually shared pathophysiology: environmental factors (e.g., normal luminal flora) inciting host immune dysregulation in genetically predisposed individuals [5]. This process involves CD4+ lymphocytes, macrophages, neutrophils, and their corresponding inflammatory mediators, with the ensuing immune response eliciting a chronic, destructive inflammatory process and associated intestinal epithelial barrier dysfunction. Of all the modes of human colorectal cancer induction (e.g., adenoma-carcinoma sequence or chromosomal instability pathway, hereditary nonpolyposis colon cancer or HNPCC, and colitis-associated), colitis-induced colorectal cancer (CAC) is unique in its pathophysiology, involving inducible nitric oxide synthase (iNOS)-mediated free radical injury in the setting of a persistently inflamed mucosa [6]. The significant impact of IBD's relapsing and remitting nature on patient well-being, coupled with its malignant potential, has led to the development of a variety of biological drugs such as antitumor necrosis factor alpha (a-TNF*α*) antibodies [7]. These have dramatically altered the outlook for patients with IBD. However, there are limitations associated with their use that include cost and significant infectious complications; these factors are now leading to a search for novel treatment options.

Curcumin is a yellow pigment found in the Indian curry spice, turmeric, and has been credited as a natural treatment for a variety of diseases. Today, this food agent is being increasingly recognized in complementary and alternative medicine (CAM) for its anti-inflammatory, antimicrobial, anticarcinogenic, antiproliferative, and free radical scavenging activity [34, 35]. In particular, its mainly intestinal localization has specified it as an agent for investigation predominantly in bowel pathology. Once overlooked, natural remedies such as curcumin are attracting increasing interest over the conventional mainstays of treatment that include 5-aminosalicylates (mesalamine), corticosteroids, immune modulators (e.g., methotrexate, azathioprine), biologic therapies such as anti-TNF*α* (infliximab, adalimumab), and anti-*α*4*β*7 antibodies (natalizumab) in IBD [32, 33]. Importantly, studies have shown IBD and CRC to be less common in developing countries than developed nations with eastern emigrants gradually assuming the western risk for disease [36, 37]. Interestingly, our studies have shown an increasing trend for IBD amongst migrants from south Asia, but colon cancer remains uncommon [38, 39]. This, again, highlights the importance of diet in these disorders and engenders greater interest in nutritional therapies such as curcumin for treatment.

## 2. Structural Anatomy of Curcumin 

First isolated in 1815, and chemically identified as a bis *α*,*β* unsaturated *β*-ketone known as diferuloylmethane [1,7-bis-(4-hydroxy-3-methoxyphenyl)-1,6-heptadiene-3,5-dione], curcumin is a hydrophobic polyphenol conferring bioactive properties to the rhizomatous herb turmeric (curcuma longa). It constitutes 5% of the plant's root [40], the remaining composition being assigned to carbohydrate, protein and essential oil contaminants. Anatomically, it is a linear diarylheptanoid comprising of 2 oxy-substituted aryl moieties held together through a 7-carbon chain. Three natural analogues of this pleiotropic compound, known collectively as curcuminoids, have been identified. Each curcuminoid contains the basic aromatic ring native to the original compound, but is differentiated by a unique methoxy group substitution in place of the aryl moieties. Curcumin is the most abundant, comprising 77%, demethoxycurcumin (17%), and finally bismethoxycurcumin (3%) [41]. Despite being structurally distinct, curcuminoids have similar biological properties. These physiological attributes are reliant on the biochemical makeup of the compound, an example being the common carbonyl and phenolic hydroxyl groups which are believed to bestow anti-inflammatory and antioxidant action to curcumin compounds, respectively. With a variable response elicited by different organ systems and their individual cellular constituents, unanimity over the most effective compound remains to be achieved, the data suggesting that curcuminoids are only synergistically more potent than any of their individual counterparts with no single analogue having greater activity compared to the next [42]. Pharmacological preparations exist as purified extracts or synthetic compositions of curcuminoids, the original powder form being cast into a convenient pill form for oral use.

A favorable safety profile relative to existing therapies adds to curcumin's appeal as a medicinal therapy. Dosage regimens up to 12 g/day are deemed well tolerated in humans [27], but achieving doses above 8 g/day has proved difficult due to the bulky structure of the compound [26]. Peak concentrations are achieved 1-2 hours after oral application dissipating to undetectable serum levels after 12 hours. Oral dosage below 4 grams has similarly rendered serum detection negligible in some studies [27]. However, availability in the gut remains comparatively higher with oral doses of even 3–6 grams showing evidence of accumulation in colorectal mucosal cells, making the bowel a prime target for therapy [31]. Human studies till date have evidenced the therapeutic efficacy of curcumin with rare patient diarrhea reported. Longer-term studies have paradoxically questioned possible tumorigenic qualities of the phytochemical at higher serum concentrations, necessitating further insight into an acceptable therapeutic range for use [43]. Studies in the Indian population have still maintained safety of lifelong curcumin ingestion up to about 100 mg/day [44], the turmeric extracts and curcumin have been tested for mutagenicity using the Ames test and have been found to be nonmutagenic [45] and the United States Food and Drug Administration has also classified the turmeric among substances Generally Recognized as Safe (GRAS).

Nevertheless, the manifold advantages of this food agent are counterbalanced by a pharmacokinetic pitfall: low bioavailability attributed to (1) poor absorption, (2) extensive first pass metabolism, (3) rapid elimination, due to hepatic glucuronidation and sulfation of the compound, and (4) poor aqueous solubility [46]. Understandably, this has accelerated the development of curcumin derivatives, which in contrast to distinct structural analogues, are in fact curcumin cores with additional functional groups (e.g., glycosyl, acetyl) [41]. One such derivative, a diphenyl difluoroketone known as EF24, has been shown to offer greater biological activity in the setting of similar toxicity as traditional curcumin [47]. Other approaches in the form of liposomal preparations, nanoparticle-based systems, and even adjuncts such as piperine and quercetin have been developed to augment curcumin's accessibility. Studies have additionally demonstrated use of heat application to curcumin as a promising means of enhancing its water solubility [48]. Metabolites of curcumin have also proved therapeutically beneficial, potentially harboring biological activity. Of these, tetrahydrocurcumin, the reduced form of curcumin, has been predominantly investigated. It lacks the yellow color and hydrophobicity of its parent compound, but despite reduced efficacy as an anti-inflammatory agent (decreased NF-*κ*B inhibition) demonstrates greater potency as an antioxidant [49].

Curcumin is known to interact with a number of targets both directly and indirectly through covalent as well as noncovalent, and hydrogen bonding. Many of these have been characterised using a computational method known as molecular docking. Curcumin's intriguing ability to bind to a multitude of different molecules is thought to be due to the fact that it contains a central beta diketone moiety, and in part due to the phenyl rings. It is capable of existing as a keto-enol tautomer, which in effect allows the molecule to undergo hydrogen bonding. Some of its most important directly interacting molecules include inflammatory molecules such as Cox-2 and TNF*α*, enzymes such as histone acetyltransferases (HATs), protein kinases such as Src and glycogen synthase kinase 3*β*, and a variety of other molecules including RNA and DNA. For example, in the case of GSK3*β*, the curcumin is bound to valine 135 and arginine 141 through hydrogen bonding (for an excellent review on this topic see [50]).

One intriguing property ascribed to curcumin is that it has epigenetic effects, which translates into an ability to affect gene regulation without actually changing the DNA sequence. In this regard it has been shown to be a stronger inhibitor of HDAC than butyrate even. The acetylation of transcription factors such as NF-*κ*B and GATA4 has also been shown to be inhibited by curcumin. One very promising area of research has addressed the role of curcumin in regulation of miRNAs. These are short lengths of nucleotides, which have the capacity to bind to the 3′ untranslated portion of messenger RNAs, and may interfere with their transcription and translation. Some of these miRNAs have been shown to play significant biological roles in the development of cancer and mediate properties such as epithelial-to-mesenchymal transition (EMT), an important attribute of cancer cells thought to be integrally involved in tissue invasion. Specifically, in this area miRNAs 21 and 200 have been demonstrated to be inhibited by curcumin [51].

## 3. Molecular Etiology and Biochemical Effects

### 3.1. *In Vitro* Studies

Chronic inflammation has been established as a noted cause of cancer [52], and it is widely accepted that treatment of IBD, together with induction of mucosal healing, also decreases the consequent risk for CRC. The molecular targets attributed to curcumin's healing qualities can be primary or secondary in nature. Primary targets are acted upon directly, while those secondarily or indirectly modified are those positively or negatively regulated according to their respective target. A key primary target in the path to mediation of the inflammatory response by curcumin is nuclear factor-kappa B (NF-*κ*B) [53]. This ubiquitous transcription factor is responsible for the regulation of expression of proinflammatory molecules including TNF*α*, IL-1, IL-6, COX-2, and 5-lipoxygenase (Table 1). NF-*κ*B activation results from the dissociation of its heterotrimeric structure (p50, p65, and I*κ*B*α*), via the phosphorylation and degradation of I*κ*B*α* through the proteosome. This results in the nuclear translocation of p50 and p65 components and initiation of transcription of inflammatory factors. Curcumin appears to reduce phosphorylation of I*κ*B*α* and its degradation by inhibiting IKK signalling [54, 55]. Inflammatory mediators arising from NF-*κ*B activation (e.g., IL-6) additionally act upon signal transducer and activator of transcription 3 (STAT-3), another pivotal cytoplasmic transcription factor, whose regulated gene products are linked with cell survival, growth, and angiogenesis. This process is reversibly inhibited by curcumin [55].

The most prevalent genetic defect associated with Crohn's disease is mutation in the intracellular pattern recognition receptor known as NOD2 (nucleotide binding oligomerization domain 2) [56]. This is part of the complex set of pathogen recognition receptors (PRRs) required for defense against mucosal pathogens. Together with NOD1, which acts as a sensor of Gram-negative bacteria, and NOD2, which likely acts as a sensor for both Gram-positive and Gram-negative, the toll-like receptors (TLRs) are key components maintaining normal gut microbial homoeostasis. Notably in this regard, genetic deletion studies indicate that both NOD1 and NOD2 knockouts have increased gastrointestinal sepsis. An interesting work indicates that curcumin is able to inhibit NOD2 signaling [57]. The relevance of this remains uncertain as it may have a positive or negative impact on chronic inflammatory disorders such as Crohn's disease.

**Figure 1:**
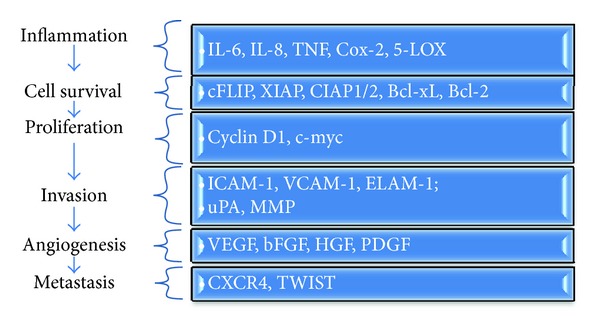
NF-*κ*B regulated molecules, directly or indirectly affected by curcumin, and their point of action in carcinogenesis.

A significant proportion of genes linked with carcinogenesis is regulated by NF-*κ*B and STAT-3; curcumin's pharmacological versatility compared to other natural anti-inflammatory agents is demonstrated by it being the sole agent capable of inhibiting both factors. One of the integral hallmarks of cancer is cancer cell survival, and curcumin is able to block this via a number of molecules with a clear involvement in survival mechanisms, several of which are NF-*κ*B dependent such as cFLIP, BCL-xL, BCL-2, XIAP, CIAP1, CIAP2, and surviving [58–63]. As these molecules may also mediate chemoresistance, it was important to show that curcumin could reverse this process through their downregulation [64]. This highlights both negative modulation of growth/proliferation pathways and galvanization of apoptotic pathways (e.g., caspase activation) [62] as an important means of inhibiting cell survival by this agent. Studies also indicate a role for curcumin in sphingomyelinase signaling with the effects on ceramide generation and ROS production mediating cell apoptosis and contributing to decreased cancer incidence [65, 66]. Cell proliferation (e.g., COX-2, cyclin D, c-myc [67–69]), angiogenesis (e.g., VEGF [70]), and invasion and metastasis (e.g., MMP-9, CXCR4, TWIST [71–73]) are also ascribed to the secondary actions of NF-*κ*b and STAT-3 (Figure 1).

A prime secondary target, indirectly mediated by curcumin through NF-*κ*B inhibition, is COX-2 [74], which is an inducible gene; its enzymatic product cycloxygenase-2 is an important intermediary in arachidonic acid metabolism. Evidence from several lines of investigation indicates that prostaglandin E2, a metabolite of arachidonic acid, plays an important role in colon cancer development [75–77]. The importance of COX-2 in colorectal cancer treatment is further substantiated by the mode of action ascribed to nonsteroidal anti-inflammatory drugs (NSAIDs) such as aspirin, which are shown to inhibit COX-2, offering a potential mechanism for reducing the risk of colon cancer [78]. As such, COX-2 inhibition hinders tumorigenesis through proapoptotic means as well as curtailing means of tumor persistence via inhibition of angiogenesis (the growth of new blood vessels) [79]. Curcumin appears to be equivalent to NSAIDs in this respect but has a more favorable toxicity profile [28], hence having significant potential for long-term bowel cancer treatment and prevention in humans.

Curcumin has been shown to inhibit vascular growth factors and zinc-dependent endopeptidases, known as matrix metalloproteinases, thus antagonizing fundamental means of tumor invasion and propagation. In a study of both primary bovine and immortalized mouse endothelial cells, curcumin inhibited endothelial cell proliferation [80]. Inhibition of angiogenesis in response to vascular endothelial growth factor (VEGF) in the human intestinal microvascular endothelium has also been demonstrated [81], together with impairment of human umbilical vein endothelial cell differentiation [82]. In human hepatocellular carcinoma cells, this phytochemical decreases hypoxia-inducible factor-1*α* (HIF1*α*), a known angiogenic transcriptional activator [83]. Additionally, alongside angiogenesis, tumour spread also requires ability for basement membrane invasiveness. Here, curcumin irreversibly inhibits and downregulates production of certain membrane-bound matrix metalloproteinases [84], with studies in human fibrosarcoma cell lines honing in on MMP-9 and MMP-2 as the main targets [85]. MMP-9 has also been reduced in human intestinal epithelial cells [64]. These collagenases are responsible for cell membrane disintegration allowing cells to invade and metastasize. Recently, the dose-dependent inhibition of MMP-3 has been identified in primary human colonic myofibroblasts isolated from patients with inflammatory bowel disease [86].

The p53 tumor suppressor gene is another molecule altered by curcumin administration. Here, divergent modification is observed. Studies in colon cancer cell lines (RKO cells) have evidenced a decline in p53 activity through impaired posttranslational modification of the gene product [87]. Nonetheless, differential activity was observed in normal B cells, which displayed minimal p53 activity. This phenomenon is echoed in studies of immature B-cell lymphoma mouse cell lines (BKS-2 and WEHI-23 cells) where inhibition of p53 was observed despite reduced cell proliferation [88]. In contrast, in the HT-29 human colon adenocarcinoma cell line, p53 induction was seen [89], supported by similar investigations in multiple other human cell studies of prostate, B-cell lymphoma and breast epithelial lineages [90]. Collectively, these studies indicate that additional factors may affect the ability of curcumin to impact on p53 function, but overall a proapoptotic role is favored.

Curcumin's unique pharmacological nature is further underscored by its effects on mitogen-activated protein kinases (MAPKs), which are important signaling intermediates for many biological processes and are activated by various receptors including growth factors, cytokines, toll-like receptors, and stress. Inhibition of this enzymatic cascade in human intestinal microvascular endothelial cells implies beneficial antiangiogenic action in response to VEGF, COX-2 and PGE2 [70]. More specifically, in colonic mucosal biopsies and colonic myofibroblasts isolated from children and adults with active IBD, curcumin also reduced p38 MAPK activation [86]. Conversely, the activation of MAPK by curcumin, for example, c-Jun N-terminal kinase (JNK) in HCT116 cells, a human colon cancer cell line [91] and p38 MAPK in primary human neutrophils [92] has also been demonstrated. The signaling mechanisms invoked by curcumin exposure proved beneficial in all respects manifesting as antineoplastic, anti-inflammatory, and antiangiogenic effects as mentioned. Generally, it is recognized that the p38MAPK and the JNKs are examples of stress-activated protein kinases, and more likely to be associated with pro-inflammatory effects and apoptosis.

The activity of PPAR*γ*, a nuclear receptor that heterodimerizes with retinoic X receptors (RXRs) and is involved in a variety of diseases, is also augmented by curcumin, leading to reduced expression of cyclin D1 and inhibition of epidermal growth factor. The resulting disruption of the cell cycle has been demonstrated with studies in Moser cells (a human colon cancer cell line) and provides another example of the remarkable versatility of this compound [93].

### 3.2. Animal Studies

Experimental studies in animal models have been widely used to demonstrate the beneficial therapeutics of curcumin in IBD and colon cancer (see Table 1). In mainly rodent models of carcinogenesis, its efficacy for colon cancer treatment and prevention has been well documented [94, 95].

Three studies, including our own, were the first to report the beneficial effects of curcumin in experimental models of inflammatory bowel disease [8, 11, 12]. These studies have been replicated by many other groups and have shown that curcumin treatment is associated with a reduction in colonic NF-*κ*B activation, Th1 cytokine profiles (e.g., IL-12), p38MAPK activation, protease activity, iNOS mRNA expression, and oxidative stresses, including MPO and lipid peroxidation [13, 15, 17]. Furthermore, it has been demonstrated that a daily diet of curcumin (30 mg/kg body weight/day) for 2 weeks in rats minimizes the ability of macrophages to generate reactive oxygen species and decreases the secretion of the lysosomal enzymes collagenase, elastase, and hyaluronidase [96]. Neutrophils represent a double-edged sword; while vital for proper immune functioning in damaged and inflamed tissues, they may also mediate epithelial injury [97]. Studies in BALB/c mice utilizing peritoneal macrophages and colonic epithelial cells show curcumin hinders neutrophil motility by antagonizing the expression and production of chemoattractant molecules, macrophage inflammatory protein 2 (MIP2), keratinocyte chemoattractant (KC), macrophage inflammatory protein 1 (MIP1), and IL-1 in the mice. In the same study, curcumin also is shown to directly block neutrophil chemotaxis [98].

Dose-dependent studies in trinitrobenzene sulfonic (TNBS) acid colitis in rats have added another layer to our understanding of curcumin bioactivity. In NKT-deficient SJL/J mice, a classic T helper cell Th1-type response is present, while BALB/c mice display both Th1/Th2 activities [20]. In this study, it was found that curcumin ameliorated disease in relation to all parameters studied only in the BALB/c mice. The reason for differing efficacies of curcumin in these two models remains unclear; however, it is suggestive of a restricted therapeutic benefit of curcumin that is dependent upon only certain immune alterations in IBD. Further dose-related studies in IL-10 knockout mouse, which simulated a Th1-type inflammation in the bowel unique to CD (versus Th2 model of UC), demonstrated a moderate dose-dependent effect detected only at the lowest dietary concentration of 0.1% [21]. Here, NF-*κ*B activation in the gut was refractory to curcumin treatment at any concentration, with a requirement for IL-10 activity to achieve this effect in epithelial cells.

From the gastrointestinal perspective, since curcumin has been shown to influence inflammation as well as cancer, and since inflammation-associated cancer is common throughout the GI tract, it was a logical extension to investigate models of colitis-associated cancer (CAC) as well as the effect of curcumin upon standard models of colon cancer, with a particular focus on inflammatory components. Hence, using repeated cycles of DSS-induced inflammation in C57BL/6 mice, one group has shown that curcumin is able to prevent the development of obvious macroscopic cancers. Only minor lesions were seen and this was associated with a reduction in the level of nuclear translocation of *β*-catenin. Interestingly, there were reductions in the levels of COX-2, i-NOS, TNF*α*, and interferon-gamma (IFN*γ*) [99].

Using a different approach and looking at APC mice, Murphy and colleagues showed that the administration of curcumin reduced the number of intestinal polyps by 75%. They were also able to show a reduction in the amount of TNF*α* in the curcumin-treated mice as well as reductions in IL-1, IL-6, and CCL2, all of which are deemed to be important players in inflammation-associated cancer [100]. 

The increasing incidence of obesity within western populations is associated with a proinflammatory state. This may be associated with an increasing incidence of cancers such as that of the colon. Using “db/db” obese mice, Kubota and colleagues were able to show that curcumin reduced the number of preneoplastic lesions [101]. In common with the other studies a reduction in the levels of TNF*α*, IL-6, and COX-2 was found. Furthermore, curcumin was also shown to reduce the levels of leptin and fat weights. 

In a more standard model of AOM-DSS-induced model of CAC, we have also shown a dramatic reduction in the number of neoplasms which was associated with impressive reductions in activity of AKT, expression of ILK, and nuclear translocations of both NF-*κ*B and *β*-catenin. Furthermore, we were able to show that reductions in the level of ILK were likely mediated through the proteosome as inhibition of this led to partial restoration of ILK levels (see [102], manuscript submitted).

Collectively, the above studies indicate an important role for a curcumin *in vivo* in several models of “inflammation-” associated cancer of the colon.

### 3.3. Human Studies

Curcumin's ability to inhibit the expression of proinflammatory stimuli has been shown clinically beneficial for patients with rheumatoid arthritis, psoriasis, postoperative inflammation, chronic anterior uveitis, and orbital inflammatory pseudotumours [103–108]. In the realm of gastrointestinal disease, curcumin shows clinical benefit in irritable bowel syndrome [109], tropical pancreatitis [110], gall bladder and biliary motility [111], gastric ulceration [112], IBD [32], and familial adenomatous polyposis coli [113]. While numerous experimental studies, both *in vitro* and colitis-simulated animal studies, have served to outline the myriad of curcumin effects in IBD and cancer, clinical studies in humans subjects remain scarce (see Table 2). Phase I clinical trials have facilitated recognition of this food agent as a safer mode of chemoprevention. Pilot dose-escalation studies in patients refractory to standard chemotherapy have demonstrated minimal side effects with doses up to 8,000 mg/day for 3 months [26]. A similar study demonstrated that a third of cancer patients given curcumin upto 2.2 g per day experienced radiologically stable disease over the course of 3 months or longer, with one patient exhibiting abatement in severity of primary colorectal cancer, indicated by a drop in levels of relevant tumor markers, thus hinting at a potential role of curcumin in tumor regression [28]. So far, there have been limited human studies with curcumin and IBD outlining its beneficial role. In a pilot study, Holt and colleagues reported its use in ulcerative proctitis and CD [32]. Five patients with ulcerative colitis were given 550 mg curcumin twice daily for 1 month followed by 550 mg three times daily for another month. All the 5 patients improved as indicated by reductions in concomitant medications in four. Similarly, five patients with CD were treated with 360 mg curcumin three times daily for 1 month with similar dosage four times daily for another two months. Here, four out of five demonstrated improvement in disease parameters as evidenced by lower Crohn's Disease Activity Index (CDAI) scores (mean reduction of 55 points), decreased ESR (mean reduction of 10 mm/hr), and reduced CRP by a mean of 0.1 mg/dL. Symptomatic improvement in these patients was also documented (*P* ≤ 0.02). In another study, Hanai et al. [33] recruited eighty-nine patients with quiescent UC in a randomized, double-blind, multicenter trial of curcumin. Forty-five patients received 1 g curcumin twice a day alongside sulfasalazine or mesalamine, with the remaining 44 patients receiving placebo plus sulfasalazine or mesalamine for a period of 6 months. Of the 45 patients, 43 maintained a satisfactory study protocol and of those 43 who received curcumin, two relapsed during 6 months of therapy (4.65%). 8 of 39 patients (20.51%) in the placebo group relapsed (*P* = 0.040). Recurrence rates evaluated on the basis of intention to treat showed a significant difference between curcumin and placebo (*P* = 0.049). Furthermore, curcumin improved both the clinical activity index (*P* = 0.038) and the endoscopic index (*P* = 0.0001) measures used to evaluate the morbidity associated with UC. The results of these studies favour curcumin as a promising and safe medication for maintaining remission in patients with quiescent UC as well as bolstering symptomatic improvement in those with proctitis and CD.

Recently, the administration of either 2 or 4 g of curcumin to smokers with evidence of aberrant crypt foci (ACFs, precursors of colonic polyps) on outcome was examined [114]. After 30 days, it was found that individuals on 4 g of curcumin but not 2 g had a 40% reduction in the number of ACFs. Although previous work indicated the possibility that curcumin could inhibit the generation of prostaglandins, this was not confirmed in this study. It was postulated that curcumin derivatives present in plasma could have accounted for these findings. Nevertheless, in conjunction with findings from the FAP study [113] this indicates an important biological effect. As the upper limit of curcumin in dosage is not yet clear, this indicates that a higher dose may be required, or more importantly, investigation into the precise mechanism involved may be informative.

Thus, in concordance with *in vitro* and animal studies, human studies serve to validate the therapeutic potential of curcumin in IBD and bowel cancer, highlighting beneficial effects in prevention, maintenance, and even possible regression of disease.

## 4. Newer Preparations of Curcumin

Given the limitations of oral dosing with curcumin to achieve adequate plasma levels, a number of different approaches have been investigated. These include liposomes, nanogels, and nanoparticles (reviewed in [115]). This essentially results in improved solubilization because of the high surface area. Preliminary work seems to indicate that this is an effective approach for the treatment of cancer, as shown in several proof-of-concept studies. For example, a particular problem with cancer chemotherapy is the development of drug resistance, through drug efflux transporters such as MDR or ABCG2, which severely limit the amount of useful drug present to destroy tumor cells; it was possible to overcome this drug resistance utilizing curcumin nanoparticles, in breast cancer [116]. 

In a study looking at the pharmacokinetics of a curcumin nanoformulation, patients with osteosarcoma given 2 to 4 g of oral solid lipid curcumin were able to achieve up to 41 ng/mL serum concentrations within four hours [117]. From a safety prospective there were no adverse effects associated with this.

These observations clearly indicate an exciting development in improving curcumin delivery for systemic use, and with further refinements, may lead to better clinical responses in both inflammatory and neoplastic disorders.

## 5. Conclusion

In evaluating the role of curcumin in gastrointestinal disease, especially IBD and colon cancer, all aspects of its use need to be considered. As outlined by countless *in vitro* and animal studies, curcumin has proven beneficial in attenuating/ameliorating biochemical, histological, and symptomatic disease parameters. Moreover, its pharmacodynamic profile as a low toxicity alternative places it on a pedestal distinct from more traditional treatments such as 5-aminosalicylates or corticosteroid therapy whose expense and/or adverse reaction profiles render them problematic [118]. The limitations of existing medicines in maintaining remission of these disorders can be a product of altered forms of immune dysregulation which remain refractory to current therapy. For example, anti-TNF*α* antibody therapy targets TNF*α* as the prime instigator of inflammation. However, as demonstrated in previous animal studies, immune alteration in IBD proves multidimensional, responsive to a certain form of treatment in some cases and not in others. Curcumin may favorably alter the “inflammatory” balance, and whether it is used as a synergistic supplement or a stand-alone therapy, or whether or not new formulations are used, the realization of a therapeutic potential (for curcumin) provides a new paradigm for gastrointestinal disease management. 

Epidemiological analysis of IBD emphasizes a distinct difference between incidence and prevalence of IBD in the West and in Asia, the latter exhibiting a relatively lower rate of both [119]. However, across Asia the incidence and prevalence of IBD have increased rapidly over the last two to four decades, these changes occurring concurrently with increased westernization of their diet. With curcumin being such a fundamental ingredient in Asian cuisine, it is not unreasonable to invoke this as a prime etiological agent contributing to the decreased prevalence of “western gastrointestinal disease” in the region, necessitating additional human studies to confirm. However, human studies are hampered by its inadequate bioavailability, attributed to poor aqueous solubility of the agent [120]. To counter this, as discussed above, new approaches to overcome curcumin's low bioavailability are being developed in the form of liposomal preparations, nanoparticle-based systems, and even adjuncts such as piperine augmenting curcumin's accessibility [25]. Thus, improved preparations for use in human patients together with the use of larger cohorts will act to validate preexisting notions regarding curcumin's utility and pave the way for greater understanding of this exotic ingredient's algorithmic role in future treatment of patients with IBD, colon cancer, and various other gastrointestinal ailments.

## Figures and Tables

**Table 1:** Curcumin: a list of animal studies demonstrating anti-inflammatory, chemopreventive, and chemotherapeutic action in gastrointestinal disease (IBD and CRC).

Author, year	Animal model	Dosage regimen	Findings
Sugimoto et al., 2002 [8]	TNBS colitis; C57BL/6 and BALB/c mice	Dietary; 0.5, 2.0, and 5.0% 7 days	Inhibits Th1 cytokine profile in CD4+ T cells by suppressing IL-12 production in macrophages; proposed mechanism: NF-*κ*B downregulation

Perkins et al., 2002 [9]	Min/+ mice and wild-type C57Bl/6J mice	Dietary; 0.1%, 0.2%, and 0.5% 15 weeks or single dose intraperitoneal injection	Concluded curcumin useful in the chemoprevention of human intestinal malignancies related to Apc mutations (advantage over NSAIDS in is its ability to decrease intestinal bleeding linked to adenoma maturation) At 0.2 and 0.5%, it reduced adenoma multiplicity by 39 and 40%, respectively Hematocrit values in untreated mice were drastically reduced

Perkins et al., 2003 [10]	Apc (Min/+) mice	Dietary; 0.2% and or aspirin (0.05%)	Aspirin and curcumin act during different “windows” of neoplastic development

Ukil et al., 2003 [11]	TNBS colitis; BALB/c mice	Dietary; 50, 100, and 300 mg/kg; 10 days before treatment and 8 days after induction	Significant reduction in neutrophil infiltration (decreased MPO activity), lipid peroxidation (decreased malondialdehyde activity), and decreased serine protease activity.; also reduction in IFN-, IL-12, iNOS mRNA expression, and NF-*κ*B

Salh et al., 2003 [12]	DNBS colitis; C3H mice	Dietary; 0.25% 5 days before treatment and 5 days after induction	NF-*κ*B inhibition; reduced MPO activity, IL-1*β* activity, and p38 MAPK activity Reduced weight loss, histological severity, and reduction in inflammatory markers

Jian et al., 2005 [13]	TNBS colitis; SPF Wistar rats	Dietary; 2.0%, 14 days	NF-*κ*B, I*κ*B, IL-1, and IL-10 improved histological score, suppression of NF-*κ*B, blockage of I*κ*B degradation, suppression of IL-1, and increase IL-10 expression

Jiang et al., 2006 [14]	TNBS colitis; Sprague-Dawley rats	30 and 60 mg/kg day, intraperitoneal injection 14 days	Reduced MPO activity, decreased COX-2, IFN- and TNF-expression, and increased PGE2 expression

Zhang et al., 2006 [15]	TNBS colitis; Sprague-Dawley rats	30 mg/kg/day, intraperitoneal injection 15 days	Reduced myeloperoxidase (MPO) activity Decreased the expression of Th1 cytokines (IL-12, IFN-gamma, TNF-alpha, and IL-1) Increased the expression of Th2 cytokines (IL-4 and IL-10) Increased proportion of IFN-gamma/IL-4 in splenocytes and circulation Improved weight loss and histological images

Venkataranganna et al., 2007 [16]	DNCB colitis; Wistar rats	Dietary; 25, 50, and 100 mg/kg 10 days	Down-regulation of iNOS and NF-*κ*B expression Decrease in MPO, LPO, and ALP activities

Camacho-Barquero et al., 2007 [17]	TNBS colitis	Dietary; 50–300 mg/kg 14 days	Reduced MPO activity and tumour necrosis factor alpha (TNF)-alpha Diminished p38 MAPK activity; decreases COX-2 and iNOS expression Reduced nitrites

Martelli et al., 2007 [18]	DNBS colitis; BALB/c mice	Dietary; 45 mg/kg; ±capsazepine intraperitoneally, (30 min before curcumin) 7 days	Reduction in the activation of p38 MAPK Down-regulation of COX-2 and iNOS expression Reductions in MPO activity and (TNF)-alpha Reduced nitrites

Deguchi et al., 2007 [19]	DSS colitis; BALB/c mice	Dietary; 2.0% wt/wt 14 days	Reduced disease activity index, histological colitis score, and MPO activity Suppressed NF-*κ*B activity

Billerey-Larmonier et al., 2008 [20]	TNBS colitis; BALB/c and SJL/J mice	Dietary; 2.0% wt/wt 9 days	BALB/c mice: curcumin significantly increased survival, prevented weight loss, and normalized disease activity SJL/J mice: curcumin demonstrated no protective effects

Larmonier et al., 2008 [21]	Specific pathogen-free wild-type 129/SvEv mice and IL-10 (−/−) mice	Dietary; 0.1–1% wt/wt 14 days	Reduced IFN-gamma and IL-12/23p40 in SPF mice (limited effects in IL-10 mice) Synergistic action of curcumin and IL-10 to inhibit NF-*κ*B

Nones et al., 2009 [22]	mdr1a −/− mice	Dietary; 0.2% 16–19 weeks	Upregulation of xenobiotic metabolism and a down-regulation of proinflammatory pathways (possibly mediated by pregnane X receptor (Pxr) and peroxisome proliferator-activated receptor alpha (PPARA) activation of retinoid X receptor (Rxr))

Lubbad et al., 2009 [23]	TNBS colitis Sprague-Dawley rats	Dietary; 100 mg/kg 5 days	Reduced MPO and MDA concentrations in colitis models Diminished TLR-4, NF-*κ*B, and MyD88 proteins in colitis models

Jia et al., 2011 [24]	DSS colitis; C57BL/6 mice	Dietary; 2% curcumin ± fish oil ± maize oil	Combined FO and curcumin suppressed NF-*κ*B, in the colon mucosa

**Table 2:** Curcumin: a list of clinical studies in humans.

Author/year	Phase	Number of patients	Dosage regimen	Findings
Trials to assess safety and pharmacokinetics				

Shoba et al., 1998 [25]	1	10	Humans: 2 g/day Mice: 2 g/kg	Piperine enhances the serum concentration, absorption, and bioavailability of curcumin in both rats and humans with no adverse effects

Chen et al., 2001 [26]	1	25	500–12,000 mg/day × 90 days	Histologic improvement of precancerous lesions; seen in 1 out of 2 patients with recently resected bladder cancer, 2 out of 7 patients with oral leukoplakia, 1 out of 6 patients with intestinal metaplasia of the stomach, 1 out of 4 patients with CIN, and 2 out of 6 patients with Bowen's disease

Lao et al., 2006 [27]	1	24	500–12,000 mg/day	Curcumin is safe and well tolerated up to 12 g/day

Author/year	Disease	Number of patients	Dosage regimen	Findings

Trials to assess efficacy in gastrointestinal disease (CRC and IBD)*				

Sharma et al., 2001 [28]	Colorectal cancer	15	36–180 mg/day × 120 days	Decrease in lymphocytic glutathione S-transferase activity Radiologically stable disease in five patients for 2–4 months of treatment Well tolerated, and dose-limiting toxicity was not observed

Sharma et al., 2004 [29]	Colorectal cancer	15	450–3600 mg/day × 120 days	Decrease in inducible PGE(2) production Daily oral dose of 3.6 g of curcumin is advocated for phase II evaluation in the prevention or treatment of cancers outside the gastrointestinal tract

Garcea et al., 2004 [30]	Liver metastasis in CRC	43	450–3600 mg/day × 7 day	Poor bioavailability following oral administration

Garcea et al., 2005 [31]	CRC	12	450–3600 mg/day × 7 days	Decreased M1G DNA adducts COX-2 levels in malignant colorectal tissue not affected by curcumin

Holt et al., 2005 [32]	IBD	10	550 mg; × 2-3/day × 60 days	All proctitis patients improved Reduced concomitant medications in four of five Crohn's disease patients Lowered CDAI scores and sedimentation rates

Hanai et al., 2006 [33]	Ulcerative colitis	89	2000 mg/day × 180 days	Maintains remission Improved both CAI (clinical activity index) and EI (endoscopic index) that is Reduced morbidity associated with UC

Ongoing trials (http://www.clinicaltrials.gov/)				

Medical University of Vienna, Department of Clinical Pharmacology [Est. date of completion N/A]	Safety and PK	N/A	Liposomal curcumin; single dose i.v infusion at 10, 20, 40, 80, 120, and 180 mg/m² over 120 minutes	Safety, tolerability, and pharmacokinetics of liposomal curcumin in phase I dose escalation study (i) to evaluate the safety and tolerability of increasing doses of intravenous liposomal curcumin in healthy subjects by means of adverse events, vital signs, and safety laboratory assessments (ii) to investigate the pharmacokinetics of increasing doses of intravenous liposomal curcumin in healthy subjects

James Graham Brown Cancer Center, US [Est. Date of completion: Jan, 2013]	Colon cancer	Recruiting	Curcumin: 3.6 g/day × 7 days Curcumin conjugated with plant exosomes tablets × 7 days	Phase I clinical trial investigating the ability of plant exosomes to deliver curcumin to normal and malignant colon tissue (i) to estimate the effect of a fixed concentration of curcumin when delivered by plant exosomes compared to oral tablets of curcumin alone

University of Leicester/University Hospitals Leicester, United Kingdom [Est. date of completion date: Jan, 2019]	Colon cancer	Recruiting		Phase I/II a study combining curcumin (curcumin C3-complex, sabinsa) with standard care FOLFOX chemotherapy in patients with inoperable colorectal cancer (i) Will focus on safety and tolerability (ii) Secondary measurements include efficacy supported by biomarker analysis

University of California, Irvine, US [Est date of completion: Dec, 2012]	Colon cancer	Estimated: 48		Phase II a trial of curcumin among patients with prevalent subclinical neoplastic lesions (aberrant crypt Foci): (i) Will determine mean percentage change in PGE2 within aberrant crypt foci (ACF) from baseline to 30 days after treatment with curcumin in current smokers.

University of North Carolina, Chapel Hill, US [Est. date of completion: March, 2012]			4 grams curcumin C3 tablet daily × 30 days	Chemoprevention of colorectal neoplasia: (i) Use of microarray analysis to identify genes that are modified by curcumin that could be used as biomarkers in future chemoprevention studies (ii) Will also evaluate tolerability and toxicity

*Patient trials have also demonstrated clinical benefit in: rheumatoid arthritis, psoriasis, postoperative inflammation, chronic anterior uveitis and orbital inflammatory pseudo-tumours, irritable bowel syndrome, tropical pancreatitis, gall bladder and biliary motility, gastric ulceration, and familial adenomatous polyposis coli.
